# Sensitive Dentine

**Published:** 1888-02

**Authors:** J. B. Willmott


ARTICLE II.

### SENSITIVE DENTINE.

BY J. B. WILLMOTT, L. D. S., D. D. S., M. D. S.*

[Read before the Seventh and Eighth District Dental Societies, Buffalo, N. Y., October, 1887.]

The subject which I have chosen, for what I fear will prove a somewhat incomplete paper, is by no means novel, but is, nevertheless, interesting to both operator and patient. So long as a large proportion of our patrons approach our rooms with feelings akin to those experienced by the victims of the inquisition in bygone ages, we, as practitioners, will be interested in the discussion of Sensitive Dentine. On this subject so much has been said and written that I cannot hope, at best, to do more than present known facts in a somewhat new aspect, and to make some deductions, which may possibly suggest a method of combating the difficulty, more intelligent, perhaps more scientific, than some which have been in use.

Though all are agreed that human dentine is endowed with the function of sensation, there is no general agreement as to the minutia of the process by which a sense of injury is conveyed to the brain so that we may take cognizance of it.

The theory elaborated by Dr. Black, in "American Dentistry," is reasonable and accounts for the phenomena observed. In his view, experiment has demonstrated that protoplasmic cells are sensitive, and manifest their sensibility in response to contact with stimulants both chemical and mechanical. The tubules of the dentine are occupied by projections from the protoplasmic odontoblast. The central end of the elongated odontoblast is in close association with the fine nerve filaments in the periphery of the pulp.

*Professor of Operative Dentistry, Royal College of Dental Surgeons of Ontario.

A fair assumption from these facts seems to be that the sense of injury experienced by the free extremity of the odonto-blasts is communicated to the nerve filaments, with which its central extremity is associated, and by these transmitted to the brain.

Whatever may be the precise *modus operandi* by which it is effected, it would seem perfectly clear, from the anatomical structure of dentine, that sensation is conveyed through, or by, the contents of the tubules, and that sensation in dentine is confined to these contents.

Though all dentine is more or less sensitive, there is a vast difference in the normal sensibility of the teeth in different individuals.

This variation is dependent on age, temperament, sex, quality of tooth tissue, and other causes, and is so great that what would be hyperæsthesia in one patient would not reach the standard of normal sensibility in another.

Ordinarily, in the discussion of the treatment of this painful condition, this fact has been overlooked. Methods of treatment which, in cases of exalted sensibility, as a pathological condition, have been entirely satisfactory, have, in apparently similar cases, proved useless and disappointing, because the condition was normal and not pathological.

Up to comparatively recent years the commonly accepted cause of hyperæsthesia of the dentine seems to have been inflammation. This theory is defended at considerable length by Dr. Taft. In the light of our present knowledge of the minute structure of dentine, as revealed by the microscope, his argument cannot be considered very conclusive. Nor has any treatment, scientifically based on the inflammatory theory, ever produced satisfactory results. Another, and more plausible suggestion, was that the dental pulp was really the seat of the exalted sensibility, and that the contents of the tubules were merely the passive instruments or agents to transmit the external impression to this central organ. Rational treatment based on this hypothesis would be the administration of such therapeutic agents as, acting on the nervous or circulatory systems, or both, should lower this exalted sensibility. The observed result of the use of nervous or arterial sedatives for this purpose has not tended to confirm the correctness of the theory.

Dr. Louis Jack has discussed the subject in the second volume of American Dentistry and concludes that "it may be considered clearly established that dentinal sensibility is attributable to the state of the tubular contents, and that it is excited into extreme manifestation by some physical irritation of the fibrillæ." The docter has only considered this sensitiveness as associated with dental caries, and attributes the physical irritation to the disintegrating process by which caries are developed. Ir is well known, however, that this condition is not confined to teeth affected by caries, and, consequently, is not always occasioned by the disintegration of dentine.

My own opinion, formed after considerable observation and study of the phenomena exhibited, and now expressed, not dogmatically but tentatively, is, that hypersensitive dentine as a pathological condition is analogous to the familiar condition known as "teeth on edge" and is produced by the same general cause, the irritation of an acid.

In a severe case of "teeth on edge," from eating sour fruit, the irritating acid is concentrated and abundant. It passes through the pores of the enamel, which is itself devoid of sensation, and acting on the peripheral extremities of the fibrillæ, causes such irritability in this tissue that the slightest impact on the external surface of the tooth, or any material elevation or depression of temperature, causes extreme discomfort. In the hyperæsthesia ordinarily observed in dental practice, in association with caries, the irritating acid is dilute and not in large quantity, so that the effect is produced slowly and requires for its manifestation greater variations of temperature, the contact of such irritating agents as sugar or salt, or some injury to the locality affected, as the cut of an excavator. The difference of the two conditions is one of degree only. In the former, the irritant being applied for a short time only, and soon becoming so diluted by the saliva as to become inert, the exalted sensibility rapidly subsides. In the latter, the irritation is persistent and the hyperæsthesia becomes chronic.

We are occasionly asked to prescribe for patients whose teeth have become so excessively sensitive that the slightest variations of temperature produce acute suffering, requiring that both food and drink be taken warm. We are frequently called upon to treat cases where the necks of the teeth have become acutely sensitive to the touch of the tooth brush or other hard substanee, and are especially so to contact with such chemical agents as sugar or salt or strong acids.

The first we assume to be due to an acid condition of the system generally, or a markedly vitiated state of the oral fluids; the last to be due to the acid secretions of the sub-mucous glands, probably associated with an acid condition of the saliva. If our theory be correct, antacid treatment, systemic or local, or both, should be effectual. In practice we find that the former condition, when not associated with other serious constitutional disturbance, will yield promptly to Potassium Bicarb. in tengrain doses three or four times daily. The latter is effectually relieved by the free use of precipitated or prepared chalk, rubbed into the interstices of the teeth and pasted around their necks on retiring at night, or by frequent rinsing of the mouth with lime water.

It is, however, with the treatment of sensitive dentine in caries that the dentist is principally concerned.

If we diagnose this as a pathological condition, the indications will be to gently remove as much of the debris as may be done without severe pain, neutralize any free acid with a drop of liquor ammonia, and fill temporarily with zinc phosphate, thus shutting out the irritant and permitting the exalted sensibility to subside.

If the sensitiveness, extreme though it be, is the normal condition of the tooth, temporary filling for a month, or for a year, could not be expected to afford any relief. The fact that the average dentist is able to discriminate with a good degree of certainty between the normal and the pathological, does not bring him much comfort. What he wants is some easily available treatment that shall promptly control either or both. For this purpose the whole materia medica has been ransacked, and on one theory or another, or on no particular theory but at hap-hazard, a large proportion of the therapeutic agents known to science have at some time been recommended and tried with such indifferent success that there is still an anxious inquiry from our patients for some relief from the tortures of dental operations.

A great deal may be accomplished by gaining the confidence of the patients—by stimulating their courage—by tact and gentleness of manner and touch, by the use only of suitable and sharp instruments, skillfully and intelligently used; but even so, there is still very much to be desired. Surely science or common sense can suggest some means to this end. Referring again to the structure of living dentine, we find the tubules occupied by fibrillæ, ready instantly to communicate the fact of any injury to their extremity. If it were possible to cause these fibrillæ to draw themselves back into the tubules so that there should be a free, unoccupied portion of the tubule which could be cut off without injury to the retracted occupant, it would seem that we had accomplished our desire. Probably not entirely; as there would still remain that part of the pain due to vibration caused by the force necessarily employed in cutting dentine, this would be slight. Is it possible to secure this retraction? Agents which stimulate contraction are at once suggested. Contraction of living tissue is, however, not a condensation of bulk but merely a change of form. As the tubules are already full and the walls are unyielding, change of form so as to produce contraction is not possible. A large percentage of the contents of the tubules is water; if a portion of this could be removed, until it could be replaced again from the central source of supply, the cell would skrink from its free end towards its central attachment.

This is doubtless what occurs when a carious tooth has been isolated and protected by the rubber dam and the free moisture in the cavity absorbed; the natural heat of the tooth slowly evaporates the water, the fibrillæ retract and the surface can be removed with less pain than when it was moist. Here, it seems to me, we have suggested to us *dehydration* as the true secret of promptly obtunding sensitive dentine whether it be normal or pathological.

There are two principal methods by which this may be accomplished: by evaporation, and by the use of agents which have a marked affinity for water. To succeed by either method it is essential to protect the cavity from moisture, not only when the dehydration is being accomplished, but until the excavation is completed. With the advent of moisture we soon have a return of sensation and that exalted by the irritation of the previous dehydration. If we purpose to dehydrate by evaporation, a good plan will be to protect the cavity, thoroughly absorb the free moisture, remove the loose debris, then saturate the cavity with absolute alcohol, and, in a minute or two, absorb it and apply a jet of warm air by one of the appliances for that purpose. In this way the water is evaporated and the fibrillæ retracted to a greater depth than by using the warm air alone. Of the available agents having a strong affinity for water, zinc chloride has long been used as an obtunder, the effect being generally ascribed to the escharotic property. The fact that the sensation returns after a brief period would seem to contradict this theory. It is more probable that its virtue is largely due to its activity as a dehydrator. If this view be correct, Dr. Jack's direction to carefully and thoroughly wash out of the cavity the dissolved zinc chloride, would appear to be a mistake. The best results will be obtained by protecting and thoroughly drying the cavity, removing the loose debris, then introducing the zinc chloride in crystals, forcing them against the walls of the cavity. When the pain has subsided, absorb the now fluid zinc chloride and carefully exclude moisture until the cavity is prepared. Whatever agent is used the same general procedure is indicated.

A preparation consisting of equal parts by weight of absolute alcohol, anhydrous glycerine and tannic acid has been used with good success, though it is doubtful if the astringent adds anything to its virtue, that depending on its dehydrating property.

What is known as Herbst's obtunder, whether so designed or not, is evidently a combination of a dehydrator, sulphuric acid, with an anæsthetic, cocaine, with a view, doubtless, to lessening the pain of the application. Having had no experience with this remedy I cannot speak from observation as to its success. As its efficiency would seem to depend on the presence of an amount of free sulphuric acid, danger to the integrity of the tooth tissue might reasonably be apprehended. What is known as Robinson's remedy—carbolate of potassium—when properly prepared is a really efficient agent. Dr Robinson's directions were to rub together equal parts of carbolic acid in crystals and potassium hydrate. This, however, results in a powdery mass very inconvenient for use. The addition of about fifteen minims of anhydrous glycerine to each dram makes a friable solid mass which can be readily applied to the cavity. That which is sold in liquid form, however valuable in the treatment of pyorrhœa alveolaris, is not the best form for use as an obtunder of sensitive dentine. In the use of this agent the same precautions are necessary in the exclusion of moisture as have already been referred to in the use of zinc chloride.

In comparison with zinc chloride the pain of application is less severe and not so long continued. My own experience would suggest that it improves with age; the chemical combination of its constituents probably requiring a considerable time to perfect. A suggestion as to the possibly far-reaching action of zinc chloride may be obtained by placing a drop of a strong solution in a considerable portion of white of egg. In the course of a few hours the coagulated mass will have extended to the diameter of probably an inch. A fragment of carbolate of potassium of similar size will, under similar circumstances, have converted a considerable portion of the albumen into a firm transparent jelly, possibly due to the abstraction of its water. Which agent is most dangerous to the integrity of the fibrillæ I am not prepared to say, but have a strong suspicion the former.

There are a number of other agents, such as dry chloride of lime, potassium carbonate, etc., which have an affinity for water, and might doubtless be used with some success. There are none, however, all things considered, equal to those already named.

Arsenious acid, for obtunding purposes, has been proven to be so dangerous to the vitality of the dental pulps, that it has ceased to be used for this purpose, and need not be here discussed.

To sum up—the points I have endeavored to make are:

1st. Excessively sensitive dentine may be either a normal or a pathological condition.

2d. As a pathological condition it is due to acid irritation.

3d. This irritation may be local and confined to the walls of the carious cavity, or it may be systemic and affect teeth otherwise healthy.

4th. This pathological condition from systemic causes may be effectually treated by antacids, and when from local causes, by the neutralizing of the debris in the cavity and temporary exclusion of the irritating agent.

5th. That exalted sensibility of dentine, whether normal or pathological, may be successfully combatted by intelligent dehydration.

6th. The treatment to be effectual must include the entire exclusion of moisture until the cavity is prepared.

7th. That the dehydrators with which I am familiar may be placed in the order of their utility as follows, viz:

(*a*) Absolute alcohol and warm air combined.

(*b*) Robinson's remedy.

(*c*) Zinc chloride in crystals.

(*d*) Alcohol, glycerine and tannin.

—*Dental Advertiser*.

